# Intramolecular carbonickelation of alkenes

**DOI:** 10.3762/bjoc.9.81

**Published:** 2013-04-12

**Authors:** Rudy Lhermet, Muriel Durandetti, Jacques Maddaluno

**Affiliations:** 1 Laboratoire COBRA, CNRS UMR 6014 & FR 3038, Université de Rouen, INSA de Rouen, 1 rue Tesnières, 76821 Mont St Aignan Cedex, France

**Keywords:** alkenes, carbometallation, carbonickelation, cyclization, Heck-type reaction, nickel catalysis

## Abstract

The efficiency of the intramolecular carbonickelation of substituted allylic ethers and amines has been studied to evaluate the influence of the groups borne by the double bond on this cyclization. The results show that when this reaction takes place, it affords only the 5-*exo-trig* cyclization products, viz. dihydrobenzofurans or indoles. Depending on the tethered heteroatom (O or N), the outcome of the cyclization differs. While allylic ethers are relatively poor substrates that undergo a side elimination and need an intracyclic double bond to proceed, allylic amines react well and afford indoline and indole derivatives. Finally, the synthesis of the trinuclear ACE core of a morphine-like skeleton was achieved by using NiBr_2_bipy catalysis.

## Introduction

Carbometalation is a reaction involving the addition of an organometallic species to a nonactivated alkene or alkyne to form a new carbon–carbon bond and generate a new organometallic entity, which may subsequently undergo synthetic transformations [1–2]. Even though these reactions have been known for over 85 years [3], they have emerged as practical organometallic tools only during the past forty years, in particular through the development of palladium chemistry [4]. The catalytic cycle starts with the oxidative addition of Pd(0) to generate a σ-arylpalladium(II), then a rapid insertion of a double or triple bond takes place [5]. This method was particularly applied in the “Mizoroki–Heck reaction” [6] for the synthesis of pharmaceutical and agrochemical intermediates using nonactivated olefins with high regio- and stereoselectivity [7]. Besides the typical intermolecular version, some intramolecular variants were developed leading to useful heterocycles [8–10]. Even if palladium is very efficient, nickel appears to be among the most promising metallic substitutes [11]. However, the tedious preparation of Ni(0) complexes such as Ni(cod)_2_ explains that nickel chemistry is hardly perceived as a realistic alternative to palladium, except in electrochemical processes [12–13]. Nevertheless, some nickel-catalyzed Heck vinylations have been recently reported on activated olefins [14–15]. Some years ago, we showed that the in situ generation of Ni(0) complexes in the presence of both the aromatic halide and the electrophile [16] represents an interesting alternative to electrochemical processes. The main advantages of the method are the use of an easily prepared Ni(II)bipy complex in combination with manganese dust as a reducing agent, which is not air sensitive, is compatible with fragile functions, and can be used in a catalytic amount. We showed that this nickel catalysis applies to cross-coupling reactions, efficiently leading to a variety of functionalized 2-arylpyridines [17]. More recently, this nickel-catalyzed reaction provided a convenient and mild method for a one-pot synthesis of substituted benzofurans, chromans and indoles by carbonickelation of alkynes [18]. We finally decided to extend the scope of this heterocyclization reaction to various nonactivated olefins in a nickel-catalyzed intramolecular base-free Heck-type coupling.

## Results and Discussion

### Scope of the reaction

**Scheme 1:**
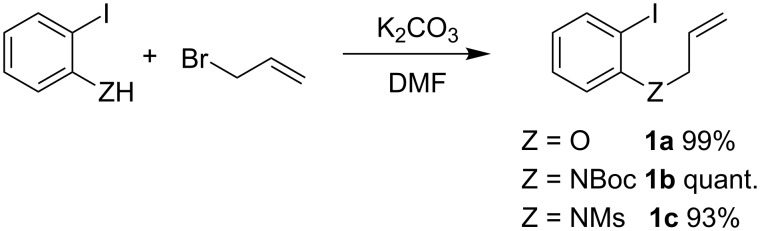
Synthesis of substrates **1a–c**.

We applied the Nickel-catalyzed intramolecular base-free Heck-type coupling to two model substrates, assumed to provide either a benzofuran or an indole core. Our first study involved the cyclization of allyl moieties such as allyl ether **1a** or *N*-allyl protected anilines **1b–c**, easily prepared by quantitative allylation of *o*-iodophenol and o-iodoanilines using allyl bromide in DMF (Scheme 1).

Following the protocol optimized for the carbonickelation of triple bonds [19], we exposed **1a–c** to a mixture containing 0.2 equiv of NiBr_2_bipy and 2 equiv of finely grown manganese in DMF containing trace amounts of trifluoroacetic acid at 50 °C (Table 1). Disappointingly, using **1a** the major new product recovered was the dimer **4a**, obtained with trace amounts of several other byproducts (Table 1, entry 1). The formation of this symmetrical dimer suggests that: (i) the oxidative addition of Ni(0) into the carbon–iodine bond leads to **1a**-Ni, which triggers a 5-*exo-trig* carbonickelation on the terminal olefin; (ii) the resulting **2a**-Ni does not undergo the expected Ni–H elimination but probably evolves by disproportionation [20] leading to alkyl_2_Ni and NiBr_2_bipy [21–22]. The subsequent reductive elimination of alkyl_2_Ni would explain the formation of the dimer **4a**.

**Table 1:** Carbonickelation of compound **1a–c**.

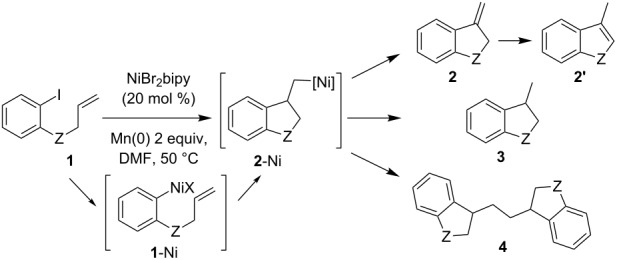			

entry	ArI	Z	ratio **2**/**2’**/**3**/**4**^a^

1	**1a**	O	ε/ε/ε/100 (26%^b^)
2	**1b**	N-Boc	0/50/50/0 (60%)
3	**1c**	N-Ms	61/26/13/0 (60%^c^)
4^d^	**1c**	N-Ms	40/22/38/0 (69%^c^)

^a^Determined by NMR, isolated yield between brackets; ^b^trace amounts of **2a**, **2a’** and **3a** were identified as well as several other byproducts; ^c^NMR yield with respect to an internal standard, based on initial aryl iodide **1c**; ^d^reaction run with 1 equiv of NiBr_2_bipy.

In contrast, the carbonickelation of **1b** led to an equimolar mixture of the expected 3-methylindole **2b’** (3-methyleneindoline **2b** rearranging into 3-methylindol **2b’**, probably during work-up) and 3-methylindoline **3b** in an overall good 60% isolated yield (Table 1, entry 2). The formation of **3** is probably due to the sluggishness of the NiH elimination, which allows for the competitive protonation of the fragile intermediate alkylnickel **2**-Ni. While the Pd-catalyzed reductive Heck reaction promoted by a hydride generated in situ is well known [23–24], the nickel-catalyzed process is likely to occur through a radical hydrogen transfer from the DMF [20,25]. The *N*-allylaniline **1c** gives the same good yield added to an attractive (**2c** + **2c’**)/**3c** ratio of 87/13 (Table 1, entry 3). Formation of the 3-methylindoline **3** is therefore disfavored when a mesyl protecting group is used instead of a carbamate. When this reaction is run with stoichiometric amounts of nickel, the reductive pathway affording indoline **3c** is slightly increased, and a (**2c** + **2c’**)/**3c** ratio of 62/38 is observed (Table 1, entry 4).

**Scheme 2:**
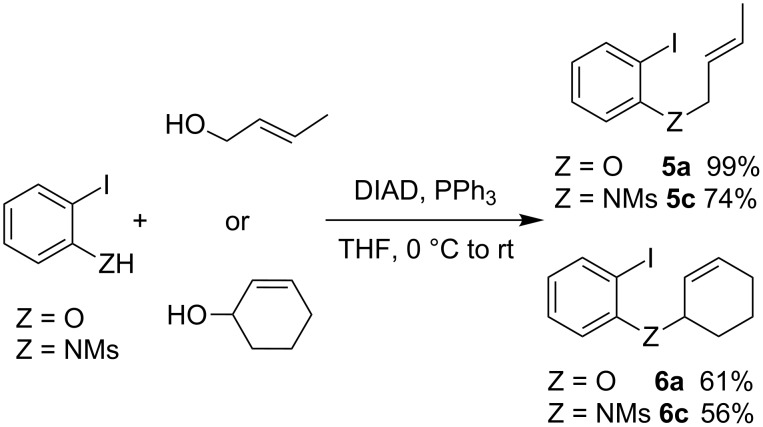
Synthesis of substrates **5a**, **5c**, **6a** and **6c**.

In conclusion to the first part of this study, the formation and cyclization of arylnickel intermediates **1**-Ni is observed in all cases. Afterward, the stability of the *exo*-methylene-nickel **2**-Ni seems to govern the formation of the cyclized product. Particularly, the dimerization of **2**-Ni threatens the synthetic utility of this reaction, as observed in the case of **1a**. In an effort to escape this pathway, we tried to stabilize **2**-Ni by using allyl moieties that would provide secondary alkylnickel intermediates. Crotyl and cyclohexenyl ethers and amines were thus employed instead of the allyl. Compounds **5** and **6** were easily prepared by a Mitsunobu condensation involving 2-iodophenol or 2-iodo-*N*-mesylaniline and crotyl alcohol or cyclohex-2-enol (Scheme 2). An *N*-mesyl derivative was retained, with a lesser amount of indoline **3** being obtained above when this protecting group was employed.

**Scheme 3:**
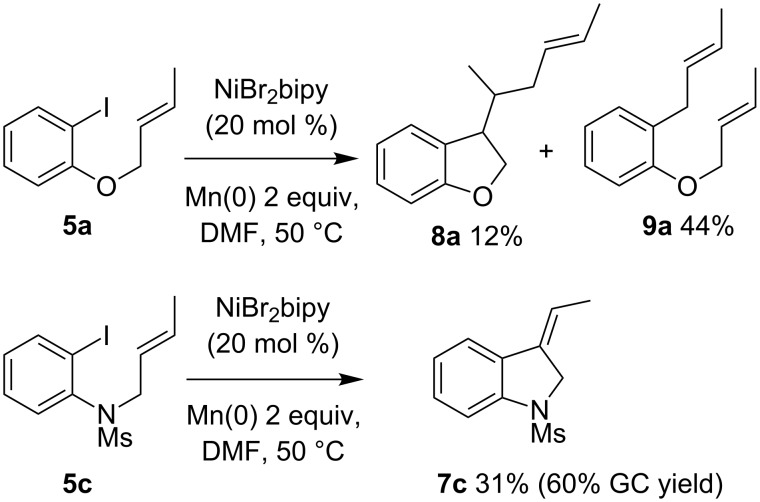
Cyclization of substrate **5a** and **5c**.

The carbonickelation protocol was applied to the cyclization of crotyl derivatives **5** (Scheme 3).

**Scheme 4:**
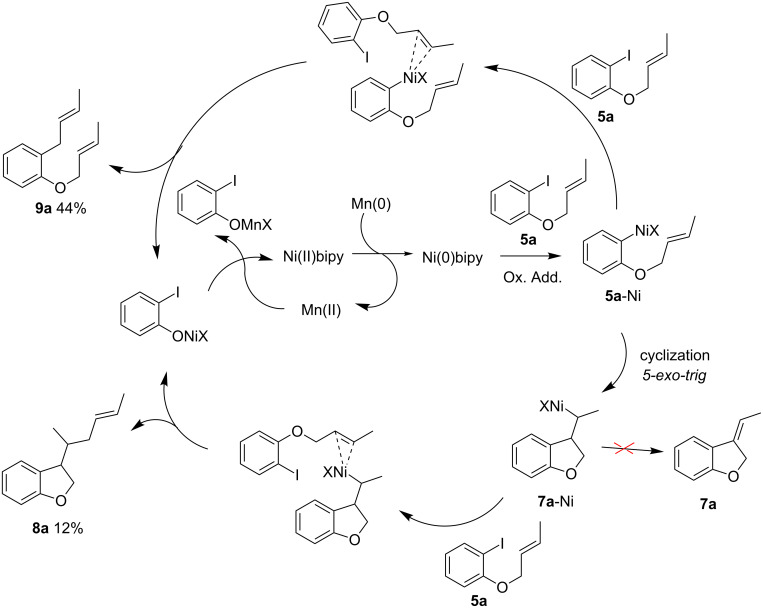
Proposed mechanism involving π-allylnickel formation.

When applied to ether **5a**, as expected, the substitution of the allyl moiety at the terminal position by a methyl tends to disfavor the dimerization of the alkylnickel intermediate, and type-**4** dimers are no longer observed. However, the expected cyclized compound **7a** is obtained only in trace amounts, with the major products isolated being compounds **8a** and **9a**. Formation of these unexpected products could result from the consecutive intermolecular reactions between **5a** and the alkylnickel **7a**-Ni or the arylnickel **5a**-Ni, respectively (Scheme 4). This reactivity is not unexpected: allyl ethers are known to be good allylating agents in the presence of nickel-bipyridine complexes [26–27]. In addition, the electroreductive allylation of aromatic or heteroaromatic halide and allylic acetate [28–29] was successfully carried out using nickel-bipyridine complexes as catalysts in DMF. More recently, Weix noted that the same reaction could be achieved under chemical conditions, always using bi-(or ter-)pyridine nickel catalysts [30]. These data explain the formation of **9a**. Finally, the **8a**/**9a** ratio suggests that **5a**-Ni can undergo two competitive pathways: the 5-*exo-trig* cyclization leading to **7a**-Ni, and the aromatic allylation affording **9a**. Based on the figures we conclude that **5a**-Ni reacts more rapidly with the allyl derivative than it cyclizes.

Under the same conditions, *N*-mesylaniline **5c** is more efficient (Scheme 3), and the expected cyclized product **7c** is obtained as the major product (60% GC yield, 31% isolated yield due to the instability of the exocyclic double bond during the purification) [31].

**Scheme 5:**
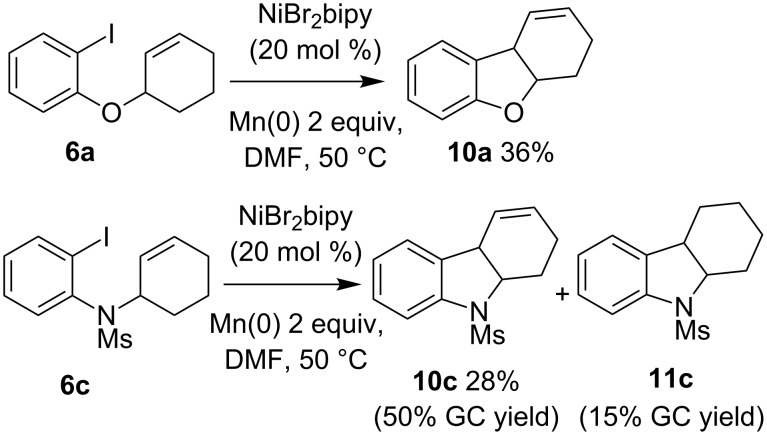
Cyclization of substrate **6a** and **6c**.

In the cyclohexenyl series, we were pleased to observe that ether **6a** affords only the benzofuran **10a**, but the conversion is limited (30% of the starting material **6a** is recovered) and the yield modest (36% isolated, 51% based on recovered material, Scheme 5).

Interestingly, the intracyclic character of the double bond in **6** appears to influence the relative kinetics of the competing reactions in favor of both the carbonickelation and the β-elimination (thus the Heck-type coupling) over the formation of a π-allyl complex that would afford allylation products such as **8** or **9**. In the aniline series, the indole **10c** (50% GC yield, 28% isolated yield) is obtained together with the indoline **11c**, in an indol/indoline **10c**/**11c** ratio of 80/20.

### Creation of an all-carbon quaternary center at a ring junction

**Scheme 6:**
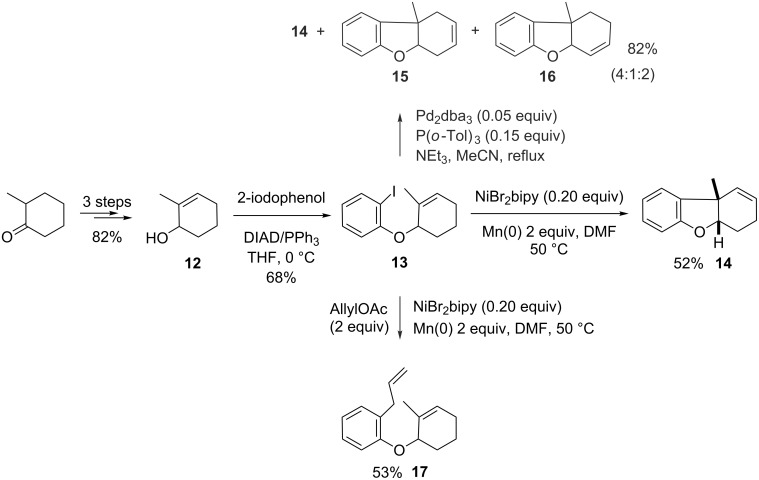
Synthesis and carbometalations of **13**.

The success of the above experiments prompted us to conduct this reaction with trisubstituted olefins, in an effort to construct tricyclic skeletons with an all-carbon quaternary center at a ring junction. This pattern is found in important natural products such as morphine whose ACE ring system exhibits a tetrahydrodibenzofuran motif with an angular ethylamino chain on C13. We thought that the nickel-catalyzed intramolecular carbometalation reaction could help to tackle the problem of the central ring (E) closure. To validate this hypothesis, we retained substrate **13** as a simplified working model. If ether **13** is little functionalized, it bears the methylvinyl moiety that is essential to the construction of the quaternary ring junction. Substrate **13** was prepared by a simple Mitsunobu condensation between 2-methylcyclohex-2-enol (**12**), a known compound prepared in three steps from commercially available 2-methylcyclohexanone and 2-iodophenol (Scheme 6). The carbonickelation protocol applied to **13** led in one hour to the expected (and sole) tricyclic product **14** in 52% isolated yield, resulting from a nickel-catalyzed intramolecular base-free Heck-type coupling and exhibiting an all-carbon quaternary center at a cis-ring junction (as established by NOESY experiments). This result, added to that obtained above with aryl ether **6a**, underlines that the endocyclic character of the unsaturation is essential to favor the Heck coupling. The carbonickelation process leads to a secondary alkylnickel intermediate, which is sufficiently stabilized to avoid the side reactions observed above. Actually, the carbonickelation remains the rate-determining step as suggested by an experiment in which allyl acetate was mixed with **13** before the addition of the catalyst (Barbier conditions). In this case, the only product was the allylated aryl derivative **17**, recovered in comparable yields, suggesting that in the presence of a good allyl donor, the σ-arylnickel undergoes an intermolecular allylation quicker than the intramolecular carbonickelation.

From a synthetic point of view, it is interesting to note that **14** was obtained as only one isomer, the double bond remaining at the location imposed by the nickel hydride elimination. In order to compare the Ni-catalyzed version to the Pd(0) one, the same reaction was run following the protocol recently described by Fukuyama [32] in the key-step of his total synthesis of morphine. Under these conditions, the cyclization took place in an excellent 82% yield after 3.5 h, but a mixture of the three regioisomers **14**–**16** was recovered (Scheme 6). By contrast, under Larock’s conditions [33] [Pd(OAc)_2_ (5 mol %), Na_2_CO_3_, DMF, 80 °C], an even higher yield (90% after 2 days) was returned but consisted of a 1:1:1 mixture of the same three products **14**–**16**. Replacing sodium carbonate by silver carbonate avoids these post-cyclization isomerizations [34–35], but the yield (52% after 16 h) was not higher than with nickel. Thus, this study suggests that a Heck-coupling reaction relying on a carbonickelation step can be considered as a useful tool in the total synthesis.

## Conclusion

In this work, we have shown that the NiBr_2_bipy complex can be used to catalyze an intramolecular Heck-type reaction in the absence of any additional base. This glove-box-free procedure occurs using 20% of NiBr_2_bipy and does not require the handling of air- or moisture-sensitive reagents. Thus, this single process gives access to a simplified model of the trinuclear ACE core of morphine. Beyond the appeal of the possible replacement of expensive palladium by cheap nickel, the absence of post-coupling isomerization of the double bond seems particularly worthy of note.

## Experimental

### General procedure for the intramolecular carbonickelation of alkenes

To a solution of aryliodide (0.5–1 mmol, 1 equiv) in anhydrous DMF (5 mL) under argon atmosphere at 50 °C is added manganese (2 equiv) followed by NiBr_2_bipy (0.2 equiv) then rapidly TFA (20 μL). The medium is vigorously stirred at 50 °C, and disappearance of the starting material is monitored by gas chromatography. The mixture is hydrolyzed with water (10 mL), diluted with Et_2_O (10 mL), and then filtered through celite. The aqueous layer is extracted with Et_2_O (2 × 10 mL), and then the combined organic layers are washed with water (3 × 10 mL) and brine (2 × 10 mL), dried over anhydrous MgSO_4_, and concentrated. The crude is purified by flash chromatography.

#### *cis*-9b-methyl-3,4,4a,9b-tetrahydrodibenzo[*b*,*d*]furan (**14**)

The compound **14** is obtained by using ether **13** (314 mg, 1 mmol), NiBr_2_bipy (75 mg, 0.2 mmol), and manganese powder (110 mg, 2 mmol) in anhydrous DMF (5 mL) following the carbonickelation procedure. The pure **14** (97 mg, 0.52 mmol, 52%) is isolated from the crude by flash chromatography on silica (2% of Et_2_O in *n*-pentane) as a colorless oil. ^1^H NMR (300 MHz, CDCl_3_) 1.41 (s, 3H), 1.78–2.03 (m, 2H), 2.18–2.31 (m, 2H), 4.62 (t, *J =* 3.6 Hz, 1H), 5.51–5.56 (m, 1H), 5.68–5.75 (m, 1H), 6.79 (dd, *J =* 8.4, 1.0 Hz, 1H), 6.87 (td, *J =* 7.2, 0.9 Hz, 1H), 7.11 (d, *J =* 7.2 Hz, 1H), 7.12 (td, *J =* 6.6, 1.2 Hz, 1H); ^13^C NMR (75 MHz, CDCl_3_) 19.4, 23.3, 25.1, 44.4, 87.8, 110.0, 120.7, 122.9, 125.5, 128.1, 132.1, 135.8, 158.9; NMR 2D NOESY: correlation between 1.41 (s, 3H) and 4.62 (t, *J =* 3.6 Hz, 1H); IR (neat): 3018, 2956, 1595, 1474, 1232, 1039 cm^−1^; MS (CI) *m*/*z*: 186 (M^+^), 171 (M − Me, base), 143, 128; HRMS (EI): calcd for (M^+^) C_13_H_14_O: 186.1045; found: 186.1049.

## Supporting Information

File 1 Experimental procedures and compound characterization.
