# Supplementary material for: Intramolecular carbonickelation of alkenes
Source: Beilstein J Org Chem. 2013 Apr 12;9:710–6. doi: 10.3762/bjoc.9.81 (PMC3678504; doi:10.3762/bjoc.9.81)
Supplement: File 1 — Experimental procedures and compound characterization. [file Beilstein_J_Org_Chem-09-710-s001.pdf]

Supporting Information
for

Intramolecular carbonickelation of alkenes

Rudy Lhermet, Muriel Durandetti* and Jacques Maddaluno*

Address: Laboratoire COBRA, CNRS UMR 6014 & FR 3038, Université de Rouen, INSA de Rouen, 1 rue Tesnières, 76821 Mont St Aignan Cedex, France

Email: Muriel Durandetti - muriel.durandetti@univ-rouen.fr; Jacques Maddaluno - jmaddalu@crihan.fr

*Corresponding author

Experimental procedures and compound characterization

General Information. GC analysis was carried out using a 24-m HP-methyl silicon capillary column. Mass spectra were recorded with a quadrupolar MS instrument coupled to a gas chromatograph. Elemental analyses were performed by the Laboratoire de Microanalyse Organique (C.N.R.S., IRCOF, Rouen). Column chromatographies were performed on standard silica gel (230–400 mesh). ^{1}H NMR spectra were recorded in $CDCl_3$ at 300 MHz, and ^{13}C NMR spectra were recorded at 75 MHz; chemical shifts (δ) are given in parts per million (ppm) and the coupling constants (*J*) in hertz. IR spectra were recorded by transmission on a IRFT spectrometer. DMF was stored under argon. THF and Et_2O were distilled from sodium/benzophenone. The catalyst precursor $NiBr_2$bipy was prepared separately according to the literature [1,2].

General procedure for the allylation reaction as exemplified by the formation of 1a: To a solution of 2-iodophenol (1.1 g, 5 mmol) in anhydrous DMF (10 mL) under an argon atmosphere is added potassium carbonate (1.38 g, 10 mmol) followed by allyl bromide (0.65 mL, 7.5 mmol). After stirring 2 h at 60 °C, the mixture is hydrolyzed with water (10 mL) and extracted with Et_2O (2 × 15 mL). Combined organic layers are washed with brine (10 mL), dried over anhydrous $MgSO_4$ and concentrated to provide the pure ether **1a** (1.39 g, 5 mmol, 99%) as a pure orange oil without further purification.

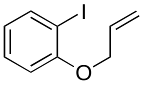

1-(Allyloxy)-2-iodobenzene (1a) (CAS registry number: 24892-63-5)

^{1}H NMR (300 MHz, $CDCl_3$) δ ppm 4.60 (dt, *J* = 4.7, 1.6 Hz, 2H), 5.32 (ddt, *J* = 10.6, 3.0, 1.6 Hz, 1H), 5.53 (ddt, *J* = 17.2, 3.0, 1.6 Hz, 1H), 6.06 (ddt, *J* = 17.2, 10.6, 4.7 Hz, 1H), 6.71 (td, *J* = 7.6, 1.3 Hz, 1H), 6.81 (dd, *J* = 8.2, 1.3 Hz, 1H), 7.28 (ddd, *J* = 8.2, 7.6, 1.6 Hz, 1H), 7.78 (dd, *J* = 7.6, 1.6 Hz, 1H); ^{13}C NMR (75 MHz, $CDCl_3$) δ ppm 69.7, 86.8, 112.6, 117.7, 122.8, 129.5, 132.6, 139.60, 157.2; IR (neat): 3060, 2864, 1581, 1470, 1246; MS (EI, 70 eV) *m/z*: 260 (M^+, base), 220 (iodophenol), 133 (M − I), 105.

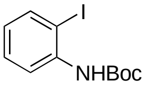

***tert*-Butyl 2-iodophenylcarbamate** (CAS registry number: 161117-84-6)

To a solution of 2-iodobenzoic acid (2.48 g, 10 mmol) in a 1:1 mixture of toluene/*t*-BuOH (60 mL) under argon atmosphere is added triethylamine (1.67 mL, 12 mmol) and diphenylphosphoryl azide (2.37 mL, 11 mmol). The reaction mixture is heated at 70 °C for 20 h, then the solvent is removed under reduced pressure and the residue dissolved in AcOEt (30 mL). The organic layer is washed with water (20 mL), saturated aqueous solution of Na_2CO_3 (20 mL) and brine (20 mL) then dried over anhydrous $MgSO_4$ and concentrated to provide the pure carbamate (2.87 g, 9.0 mmol, 90%) as a colorless oil without further purification. ^{1}H NMR (300 MHz, $CDCl_3$) δ ppm 1.54 (s, 9H), 6.76 (t, *J* = 7.6, 1H), 6.81 (s, 1H), 7.31 (t, *J* = 7.6 Hz, 1H), 7.65 (d, *J* = 7.9 Hz, 1H), 7.82 (d, *J* = 8.0 Hz, 1H).

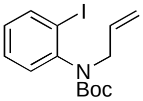

***tert*-Butyl allyl(2-iodophenyl)carbamate (1b)** (CAS registry number: 161646-50-0)

To a solution of previous carbamate (957 mg, 3 mmol) in DMF (20 mL) under argon atmosphere at 0 °C is carefully added sodium hydride (132 mg, 3.3 mmol). The mixture is stirred for 30 min

at 0 C before adding allyl bromide (311 μL, 3.6 mmol) and then for 4 h at room temperature. After hydrolysis by the addition of water (10 mL) and extraction of the aqueous layer with Et_2O (3 × 10 mL), the combined organic layers are washed with water (20 mL) and brine (20 mL), dried over anhydrous $MgSO_4$ and concentrated. The pure **1b** (1.10 g, 3 mmol) is obtained quantitatively as yellow solid without further purification. ^{1}H NMR (300 MHz, $CDCl_3$) δ ppm 1.35 (s, 6H), 1.53 (s, 3H), 3.76 (dd, *J* = 7.1, 15.1 Hz, 1H), 4.49 (dd, *J* = 5.6, 15.2 Hz, 1H), 5.02–5.15 (m, 2H), 5.85–6.03 (m, 1H), 6.97 (t, *J* = 7.4 Hz, 1H), 7.12 (d, *J* = 7.4 Hz, 1H), 7.31 (t, *J* = 7.1 Hz, 1H), 7.85 (d, *J* = 7.4 Hz, 1H); ^{13}C NMR (75 MHz, $CDCl_3$ δ ppm 28.3 (3C), 52.1, 80.4, 100.7, 118.0, 128.7, 128.8, 130.0, 133.7, 139.4, 144.3, 153.9.

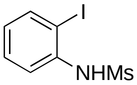

***N*-(2-Iodophenyl)methanesulfonamide** (CAS registry number: 116547-92-3)

To a solution of 2-iodoaniline (7,0 g, 31.9 mmol) in anhydrous pyridine (50 mL) under an argon atmosphere at 0 °C is added DMAP (414 mg, 3.38 mmol) and methanesulfonyl chloride (2.8 mL, 36.3 mmol). The mixture is stirred 1 h at 0 °C then hydrolyzed with aqueous solution of HCl 2 M (50 mL) and diluted with CH_2Cl_2. The organic layer is washed with HCl 2 M (2 × 20 mL) then the combined aqueous layers are extracted with CH_2Cl_2 (3 × 20 mL). After extraction of organic layers with aqueous solution of NaOH 0.5 M (3 × 20 mL), the sulfonamide is precipitated by the addition of 12 M solution of HCl in the aqueous layer, then extracted with CH_2Cl_2 (3 × 20 mL) and dried over anhydrous $MgSO_4$. Removal of volatiles provided the pure sulfonamide (8.37 g, 28.2 mmol, 89%) as a white solid. ^{1}H NMR (300 MHz, $CDCl_3$) δ ppm 3.01 (s, 3H), 6.70 (s, 1H), 6.94 (td, *J* = 7.9, 1.2 Hz, 1H), 7.38 (td, *J* = 7.5, 1.1 Hz, 1H), 7.65 (dd, *J* = 7.9, 1.5 Hz, 1H), 7.82 (dd, *J* = 7.9, 1.27 Hz, 1H); ^{13}C NMR (75 MHz, $CDCl_3$) δ ppm 40.3, 92.4, 122.7, 127.4, 129.94, 137.6, 139.5; mp: 93–94 °C.

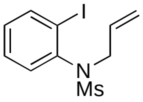

***N*-Allyl-*N*-(2-iodophenyl)methanesulfonamide (1c)** (CAS registry number: 591219-79-3)

Compound **1c** is obtained using previous sulfonamide (1.49 g, 5 mmol), potassium carbonate (1.38 g, 10 mmol) and allyl bromide (0.52 mL, 6 mmol) in anhydrous DMF (50 mL) following the allylation reaction procedure. The crude provided **1c** (1.55 g, 4.63 mmol, 93%) as a pale yellow crystals without further purification. ^{1}H NMR (300 MHz, $CDCl_3$) δ ppm 3.09 (s, 3H), 4.01 (dd, *J* = 15.0, 7.3 Hz, 1H), 4.39 (dd, *J* = 15.0, 6.0 Hz, 1H), 5.09 (d, *J* = 15.8 Hz, 1H), 5.13

(d, J = 9.8 Hz, 1H), 5.85–6.03 (m, 1H), 7.00–7.09 (m, 1H), 7.33–7.39 (m, 2H), 7.92 (dd, J = 7.8 0.9 Hz1H); ^{13}C NMR (75 MHz, $CDCl_3$) δ ppm 41.4, 54.2, 101.7, 120.0, 129.2, 130.2, 132.52, 132.54, 140.3, 140.9; mp: 70–71 °C.

General procedure for the intramolecular carbonickelation of alkenes

To a solution of aryliodide (0.5–1 mmol, 1 equiv) in anhydrous DMF (5 mL) under argon atmosphere at 50 °C is added manganese (2 equiv) followed by $NiBr_2$bipy (0.2 equiv) then rapidly TFA (20 µL). The medium is vigorously stirred at 50 °C and the disappearance of the starting material is monitored by gas chromatography. The mixture is hydrolyzed with water (10 mL), diluted with Et_2O (10 mL), and then filtered through celite. The aqueous layer is extracted with Et_2O (2 × 10 mL), and then the combined organic layers are washed with water (3 × 10 mL) and brine (2 × 10 mL), dried over anhydrous $MgSO_4$ and concentrated. The crude is purified by flash chromatography.

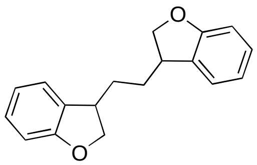

1,2-Bis(2,3-dihydrobenzofuran-3-yl)ethane (4a) (CAS registry number: 213623-05-3)

Dimer **4a** is obtained using the ether **1a** (130 mg, 0.5 mmol), $NiBr_2$bipy (37 mg, 0.1 mmol), and manganese powder (55 mg, 1 mmol) in anhydrous DMF (5 mL) following the carbonickelation procedure. The pure **4a** (17 mg, 0.064 mmol, 26%) is isolated from the crude by flash chromatography on silica (10% of Et_2O in *n*-pentane) as white crystals. ^{1}H NMR (300 MHz, $CDCl_3$) δ ppm 1.49–1.96 (m, 4H), 3.42 (bs, 2H), 4.18–4.23 (m, 2H), 4.59–4.65 (m, 2H), 6.91–6.71 (m, 4H), 7.18–7.06 (m, 4H); ^{13}C NMR (75 MHz, $CDCl_3$) δ ppm 32.3 (2C), 32.4 (2C), 42.0 (2C), 76.7 (2C), 109.8 (2C), 120.6 (2C), 124.4 (2C), 128.5 (2C), 130.5 (2C), 160.0 (2C); MS (EI, 70 eV) *m/z*: 266 (M^+), 132 (monomer), 119 (dihydrobenzofurane, base).

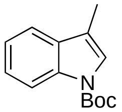

***tert*-Butyl 3-methyl-1*H*-indole-1-carboxylate (2’b)** (CAS registry number: 89378-43-8)

Compounds **2’b** and **3b** are obtained in a 1:1 mixture using carbamate **1b** (359 mg, 1 mmol), $NiBr_2$bipy (75 mg, 0.2 mmol), and manganese powder (110 mg, 2 mmol) in anhydrous DMF (5 mL) following the carbonickelation procedure. The pure **2’b** and **3b** (139 mg, 0.6 mmol, 60%) is isolated from the crude by flash chromatography on silica (10% of Et_2O in *n*-pentane) as a

colorless oil. ^{1}H NMR (300 MHz, $CDCl_3$) δ ppm 1.65 (s, 9H), 2.26 (s, 3H), 7.25 (td, *J* = 7.2 1.1Hz, 1H), 7.32 (td, *J* = 7.6 1.1Hz, 1H), 7.34 (bs, 1H), 7.50 (dd, *J* = 7.6 1.1Hz, 1H), 8.12 (bd, *J* = 7.2Hz, 1H); ^{13}C NMR (75 MHz, $CDCl_3$) δ ppm 9.8, 28.4 (3C), 83.3, 115.2, 116.5, 119.0, 122.4, 122.9, 124.3, 131.6, 135.6, 150.0; MS (EI, 70 eV) *m/z*: 231 (M^+), 175 (M − *t*-Bu, base), 130, 57.

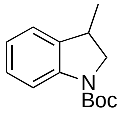
***tert*-Butyl 3-methylindoline-1-carboxylate (3b)** (CAS registry number: 161646-57-7)

^{1}H NMR (300 MHz, $CDCl_3$) δ ppm 1.32 (d, *J* = 6.8 Hz, 3H), 1.57 (s, 9H), 3.32–3.56 (m, 2H), 4.14 (m, 1H), 6.95 (td, *J* = 7.4, 1.0 Hz, 1H), 7.11–7.19 (m, 2H), 7.8 (bs, 1H); ^{13}C NMR (75 MHz, $CDCl_3$) δ ppm 20.4, 28.6 (3C), 34.2, 56.7, 80.7 (broad), 114.7, 122.3, 123.7, 127.6, 136.8 (broad), 143.0 (broad), 152.7; MS (EI, 70 eV) *m/z*: 233 (M^+), 177 (M – *t*-Bu, base), 162, 130, 118.

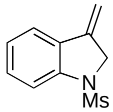
3-Methylene-1-(methylsulfonyl)indoline (2c) (CAS registry number: 118481-28-0)

Compounds **2c**, **2'c** and **3c** are obtained in a 61:26:13 mixture (difficult to separate) using sulfonamide **1c** (337 mg, 1 mmol), $NiBr_2$bipy (75 mg, 0.2 mmol), and manganese powder (110 mg, 2 mmol) in anhydrous DMF (5 mL) following the catalytic carbonickelation procedure. The pure **2c** (28 mg, 0.134 mmol, 13%) is isolated from the crude by flash chromatography on silica (15% of AcOEt in *n*-pentane) as a colorless oil. ^{1}H NMR (300 MHz, $CDCl_3$) δ ppm 2.88 (s, 3H), 4.63 (t, *J* = 2.6 Hz, 2H), 5.13 (t, *J* = 2.6 Hz, 1H), 5.53 (t, *J* = 2.6 Hz, 1H), 7.09 (t, *J* = 7.5 Hz, 1H), 7.29 (t, *J* = 7.8 Hz, 1H), 7.45–7.52 (m, 2H); ^{13}C NMR (75 MHz, $CDCl_3$) δ ppm 35.3, 55.5, 103.1, 114.6, 121.5, 124.2, 129.9, 130.6, 140.2, 144.5.

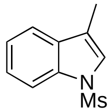
3-Methyl-1-(methylsulfonyl)-1*H*-indole (2'c) (CAS registry number: 108665-97-0)

2'c (7.5 mg, 0.072 mmol, 7%) is isolated from the crude by flash chromatography on silica (15% of AcOEt in *n*-pentane) as a colorless oil. ^{1}H NMR (300 MHz, $CDCl_3$) δ ppm 2.30 (s, 3H), 3.03 (s, 3H), 7.20 (s, 1H), 7.24-7.29 (m, 2H), 7.56 (dd, *J* = 7.2 1.2 Hz, 1H), 7.90 (d, *J* = 7.7 Hz, 1H).

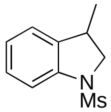
3-Methyl-1-(methylsulfonyl)indoline (3c) (CAS registry number: 115875-77-9)

^{1}H NMR (300 MHz, $CDCl_3$) δ ppm 1.36 (d, *J* = 6 Hz, 3H), 3.00 (s, 3H), 3.45–3.50 (m, 2H), 4.01–4.05 (m, 1H), 7.12-7.16 (m, 1H), 7.25-7.33 (m, 2H), 7.42-7.48 (m, 1H).

General procedure for the Mitsunobu reaction as exemplified by the formation of 5a: To a solution of 6 mmol of iodophenol (1.32 g), 7.2 mmol of triphenylphosphine (1.89 g), and 7.2 mmol of 3-methylbut-2-enol (0.61 mL) in 30 mL of distilled THF at 0 °C under argon was added dropwise 7.2 mmol of DIAD (1.43 mL). The solution was warmed to room temperature, stirred for 30 minutes and washed by adding NaOH 0.5 M (30 mL). The mixture was diluted by Et_2O (30 mL) and washed again by NaOH 0.5 M (30 mL) and then water (30 mL). The combined organic layers were dried over $MgSO_4$ and concentrated. Pentane (30 mL) was added to the residue to precipitate triphenylphosphine oxide. After filtration the oil obtained was purified by column chromatography eluted with 1% of Et_2O in *n*-pentane to give 1.62 g of the desired compound **5a** (5.96 mmol, 99%) as a pale yellow oil.

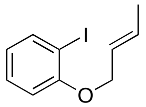
1-(But-2-enyloxy)-2-iodobenzene (5a) (CAS registry number: 120568-90-3)

^{1}H NMR (300 MHz, $CDCl_3$) δ ppm 1.77 (dd, *J* = 6.7, 1.4 Hz, 3H), 4.49–4.57 (m, 2H), 5.66–5.80 (m, 1H), 5.84–5.98 (m, 1H), 6.70 (td, *J*=7.5, 1.3 Hz, 1H), 6.82 (dd, *J* = 8.2, 1.2 Hz, 1H), 7.28 (ddd, *J* = 8.2, 7.5, 1.6 Hz, 1H), 7.77 (dd, *J* = 7.5, 1.6 Hz, 1H); ^{13}C NMR (75 MHz, $CDCl_3$) δ ppm 18.1, 69.9, 86.9, 112.7, 122.6, 125.7, 129.5, 130.3, 139.6, 157.4; IR (neat): 3059, 2914, 2854, 1568, 1743, 1242; MS (EI, 70 eV) *m/z*: 274 (M^+), 220 (iodophenol, base), 155, 55 (side chain).

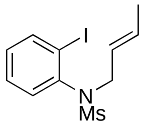
(*E*)-*N*-(But-2-enyl)-*N*-(2-iodophenyl)methanesulfonamide (5c) (CAS registry number: 591219-77-1)

5c is obtained using *N*-(2-iodophenyl)methanesulfonamide (1.49 g, 5 mmol), crotyl alcohol (0.56 mL, 6 mmol), triphenylphosphine (1.57 g, 6 mmol) and DIAD (1.19 mL, 6 mmol) in anhydrous THF (50 mL) following the Mitsunobu procedure. The pure **5c** (1.26 g, 3.7 mmol, 74%) is isolated from the crude by flash chromatography on silica (15% of AcOEt in *n*-pentane) as

colorless crystals. ^{1}H NMR (300 MHz, $CDCl_3$) δ ppm 1.61 (d, *J* = 5.9 Hz, 3H), 3.05 (s, 3H), 3.92 (dd, *J* = 14.7, 7.0 Hz, 1H), 4.28 (dd, *J* = 14.7, 5.8 Hz, 1H), 5.39–5.65 (m, 2H), 6.98–7.08 (m, 1H), 7.28-7.41 (m, 2H), 7.89 (d, *J* = 8.1 Hz, 1H); ^{13}C NMR (75 MHz, $CDCl_3$) δ ppm 17.2, 40.6, 52.9, 102.0, 124.5, 128.6, 129.6, 130.9, 131.4, 139.5, 140.5; MS (EI, 70 eV) *m/z*: 351 (M^+), 297 (M − crotyl), 218, 203, 161, 144 (base), 77, 55; mp: 58–59 °C.

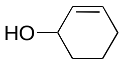

Cyclohex-2-enol (CAS registry number: 822-67-3)

To a solution of cerium trichloride heptahydrate (5.60 g, 15 mmol) in MeOH (15 mL) is added 2-cyclohexen-1-one (1.45 mL, 15 mmol), followed by sodium borohydride (567 mg, 15 mmol) portionwise over 2 min. The white precipitate is stirred 4 min at room temperature, then hydrolyzed with water (15 mL). The aqueous layer is extracted with Et_2O (2 × 15 mL), and the combined organic layers are washed with brine (15 mL), dried over anhydrous $MgSO_4$, and concentrated. The crude provided cyclohex-2-enol (1.28 g, 13.1 mmol, 87%) as a pure colorless oil without further purification. ^{1}H NMR (300 MHz, $CDCl_3$) δ ppm 1.52–2.11 (m, 7H), 4.16–4.23 (m, 1H), 5.69–5.79 (m, 1H), 5.79–5.90 (m, 1H); ^{13}C NMR (75 MHz, $CDCl_3$) δ ppm 19.0, 25.1, 32.0, 65.5, 130.0, 130.5.

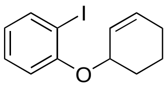

1-(Cyclohex-2-enyloxy)-2-iodobenzene (6a) (CAS registry number: 122776-64-1)

Ether **6a** is obtained using 2-iodophenol (2.42 g, 11 mmol), cyclohex-2-enol (1.28 g, 13.1 mmol), triphenylphosphine (3.42 g, 13.1 mmol), and DIAD (2.59 mL, 13.1 mmol) in anhydrous THF (50 mL) following the Mitsunobu procedure. The pure **6a** (2.03g, 6.8 mmol, 61%) is isolated from the crude by flash chromatography on silica (1% of Et_2O in *n*-pentane) as a colorless oil. ^{1}H NMR (300 MHz, $CDCl_3$) δ ppm 1.55–2.24 (m, 6H), 4.72-4.85 (m, 1H), 5.85–5.94 (m, 1H), 5.95–6.03 (m, 1H), 6.70 (td, *J* = 7.5, 1.4 Hz, 1H), 6.89 (dd, *J* = 8.3, 1.4 Hz, 1H), 7.27 (ddd, *J* = 8.3, 7.5, 1.6 Hz, 1H), 7.78 (dd, *J* = 7.5, 1.6 Hz, 1H); ^{13}C NMR (75 MHz, $CDCl_3$) δ ppm 19.0, 25.2, 28.5, 72.79, 88.6, 114.4, 122.7, 125.9, 129.4, 132.7, 139.7, 156.9; IR (neat):3028, 2958, 2864, 1580, 1467, 1241; MS (EI, 70 eV) *m/z*: 300 (M^+), 220 (iodophenol, base), 191.

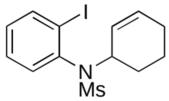 **N-(Cyclohex-2-enyl)-N-(2-iodophenyl)methanesulfonamide (6c)** (CAS registry number: 270925-87-6)

6c is obtained using *N*-(2-iodophenyl)methanesulfonamide (1.49 g, 5 mmol), cyclohex-2-enol (0.59 mL, 6 mmol), triphenylphosphine (1.57 g, 6 mmol) and DIAD (1.19 mL, 6 mmol) in anhydrous THF (50 mL) following the Mitsunobu procedure. The pure **6c** (1.6 g, 2.8 mmol, 56%) is isolated from the crude by flash chromatography on silica (15% of AcOEt in *n*-pentane) as a white solid. ^{1}H NMR (300 MHz, $CDCl_3$) δ ppm 1.37–2.34 (m, 6H), 3.16 (s, 3H), 4.59–4.79 (m, 1H), 5.83–5.92 (m, 1H), 5.99–6.03 (m, 1H), 7.06 (ddd, *J* = 7.8, 7.3, 1.8 Hz, 1H), 7.32–7.46 (m, 2H), 7.95 (dd, *J* = 7.9, 1.8 Hz, 1H); ^{13}C NMR (75 MHz, $CDCl_3$) δ ppm 21.5, 24.4, 28.0, 42.0, 58.8, 105.2, 128.2, 129.0, 130.2, 132.2, 133.1, 139.8, 140.5; mp: 93–94 °C.

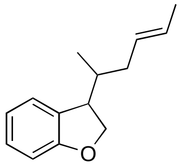 **3-(Hex-4-en-2-yl)-2,3-dihydrobenzofuran (8a)**

Dihydrobenzofurane **8a** is obtained using the ether **5a** (274 mg, 1 mmol), $NiBr_2$bipy (74 mg, 0.2 mmol), and manganese powder (110 mg, 2 mmol) in anhydrous DMF (5 mL) following the carbonickelation procedure. The pure **8a** (12 mg, 0.06 mmol, 12%) is isolated from the crude by flash chromatography on silica (1% of Et_2O in *n*-pentane) as a colorless oil. ^{1}H NMR (300 MHz, $CDCl_3$) δ ppm 0.78 (d, *J* = 6.5 Hz, 3H), 1.67 (d, *J* = 4.6 Hz, 3H), 1.85–1.93 (m, 2H), 1.99–2.10 (m, 1H), 3.46–3.56 (m, 1H), 4.37 (dd, *J*=9.3, 6.5 Hz, 1H), 4.48 (dd, *J* = 9.3, 6.5 Hz, 1H), 5.40–5.47 (m, 2H), 6.77 (d, *J* = 8.0 Hz, 1H), 6.85 (t, *J* = 7.4, Hz, 1H), 7.18–7.07 (m, 2H); MS (EI, 70 eV) *m/z*: 202 (M^+), 187 (M – CH_3), 119 (dihydrobenzofurane, base).

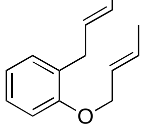 **1-(But-2-enyl)-2-(but-2-enyloxy)benzene (9a)**

The pure **9a** (44 mg, 0.22 mmol, 44%) is isolated from the crude by flash chromatography on silica (1% of Et_2O in *n*-pentane) as a colorless oil. ^{1}H NMR (300 MHz, $CDCl_3$) δ ppm 1.69–1.77 (m, 6H), 3.34 (d, *J* = 6.3 Hz, 2H), 4.48 (d, *J* = 5.5 Hz, 2H), 5.43–5.96 (m, 4H), 6.85 (d, *J* = 8.2 Hz, 1H), 6.90 (t, *J* = 7.4 Hz, 1H), 7.11–7.21 (m, 2H); ^{13}C NMR (75 MHz, $CDCl_3$) δ ppm 18.0,

18.1, 33.2, 68.9, 111.9, 120.6, 126.1, 126.6, 127.1, 129.4, 129.5, 129.7, 130.0, 156.5; MS (EI, 70 eV) *m/z*: 202 (M^+), 148 (M-side chain, base), 133, 107.

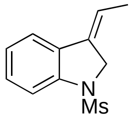

(*Z*)-3-Ethylidene-1-(methylsulfonyl)indoline (7c) (CAS registry number: 850668-94-9)

The pure (*Z*)-**7c** is obtained using sulfonamide **5c** (351 mg, 1 mmol), $NiBr_2$bipy (75 mg, 0.2 mmol), and manganese powder (110 mg, 2 mmol) in anhydrous DMF (5 mL) following the carbonickelation procedure. The mixture (68 mg, 0.3 mmol, 31%) is isolated from the crude by flash chromatography on silica (15% of AcOEt in *n*-pentane) as an orange oil. ^{1}H NMR (300 MHz, $CDCl_3$) δ ppm 1.77 (dt, *J* = 7.1, 1.9 Hz, 3H), 2.86 (s, 3H), 4.53–4.58 (m, 2H), 5.92–6.05 (m, 1H), 7.02 (t, *J* = 7.7 Hz, 1H), 7.18 (t, *J* = 7.7 Hz, 1H), 7.37 (d, *J* = 7.7 Hz, 1H), 7.45 (d, *J*=8.0 Hz, 1H); ^{13}C NMR (75 MHz, $CDCl_3$) δ ppm 14.8, 35.0, 53.6, 114.3, 114.5, 120.3, 123.9, 129.1, 130.2, 132.7, 143.5.

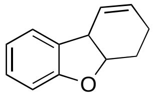

3,4,4a,9b-Tetrahydrodibenzo[*b,d*]furan (10a) (CAS registry number: 680585-93-7)

10a is obtained using the ether **6a** (600 mg, 2 mmol), $NiBr_2$bipy (150 mg, 0.4 mmol), and manganese powder (220 mg, 4 mmol) in anhydrous DMF (10 mL) following the carbonickelation procedure. The pure **10a** (125 mg, 0.76 mmol, 36%) is isolated from the crude by flash chromatography on silica (1% of Et_2O in *n*-pentane) as a colorless oil. ^{1}H NMR (300 MHz, $CDCl_3$) δ ppm 1.83–2.29 (m, 4H), 3.83 (d, *J* = 7.0 Hz, 1H), 4.96–5.06 (m, 1H), 5.71–5.94 (m, 2H), 6.81 (d, *J* = 8.0 Hz, 1H), 6.86 (t, *J*=7.4 Hz, 1H), 7.10–7.16 (m, 1H), 7.19 (d, *J* = 7.4 Hz, 1H); ^{13}C NMR (75 MHz, $CDCl_3$) δ ppm 19.7, 25.1, 41.2, 81.5, 110.0, 120.6, 124.5, 126.4, 127.9, 128.2, 131.3, 159.3; MS (EI, 70 eV) *m/z*: 172 (M^+, base), 157 ($M - CH_3$), 144, 131.

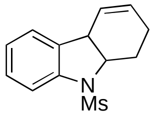

9-(Methylsulfonyl)-2,4a,9,9a-tetrahydro-1*H*-carbazole (10c)

Compound **10c** is obtained using aniline **6c** (377 mg, 1 mmol), $NiBr_2$bipy (75 mg, 0.2 mmol), and manganese powder (110 mg, 2 mmol) in anhydrous DMF (5 mL) following the carbonickelation procedure. The pure **10c** (70 mg, 0.28 mmol, 28%) is isolated from the crude by flash chromatography on silica (15% of AcOEt in *n*-pentane) as an orange solid. ^{1}H NMR (300 MHz, $CDCl_3$) δ ppm 1.66–1.83 (m, 1H), 2.01-2.05 (m, 3H), 2.92 (s, 3H), 3.97 (d, *J* = 8.3 Hz,

1H), 4.44–4.57 (m, 1H), 5.87–6.04 (m, 2H), 7.07 (t, *J*= 7.4 Hz, 1H), 7.15–7.24 (m, 2H), 7.40 (d, *J* = 8.4 Hz, 1H); ^{13}C NMR (75 MHz, $CDCl_3$) δ ppm 22.4, 26.3, 37.6, 41.0, 62.7, 115.8, 124.3, 124.5, 124.6, 128.2, 129.1, 135.0, 140.3; mp: 104–105 °C.

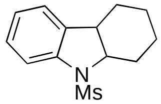

9-(Methylsulfonyl)-5,6,7,8,8a,9-hexahydro-4b*H*-carbazole (11c) (CAS registry number: 680585-93-7)

^{1}H NMR (300 MHz, $CDCl_3$) δ ppm 1.23–1.30 (m, 2H), 1.57–1.82 (m, 4H), 1.92–2.11 (m, 1H), 2.25–2.35 (m, 1H), 3.00 (s, 3H), 3.36–3.54 (m, 1H), 4.26–4.45 (m, 1H), 7.07 (t, *J* = 7.4 Hz, 1H), 7.25–7.35 (m, 2H), 7.40 (d, *J* = 8.4 Hz, 1H).

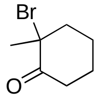

2-Bromo-2-methylcyclohexanone

To a solution of *N*-bromosuccinimide (10.68 g, 60 mmol) in CCl_4 (200 mL) under argon atmosphere is added dropwise 2-methylcyclohexanone (6.07 mL, 50 mmol). The mixture is heated under reflux for 2 h 30 min then cooled down to room temperature and concentrated to provide a yellow residue. After addition of *n*-pentane (50 mL), the solution is filtrated through Celite and the organic layer is concentrated to afford 2-bromo-2-methylcyclohexanone as a yellow oil, which is not purified. ^{1}H NMR (300 MHz, $CDCl_3$) δ ppm 1.61 (td, *J* = 14.5, 3.9 Hz, 1H), 1.77–1.80 (m, 2H), 1.81 (s, 3H), 1.97–2.17 (m, 2H), 2.29–2.43 (m, 2H), 3.21 (dt, *J*=14.5, 6.2, Hz 1H); MS (EI, 70 eV) *m/z*: 190–192 (M^+), 146–148, 111 (M-Br), 83, 55 (base).

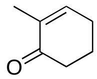

2-Methylcyclohex-2-enone (CAS registry number: 1121-18-2)

To the crude solution of 2-bromo-2-methylcyclohexanone in DMF (100 mL) under argon atmosphere is added potassium carbonate (20.73 g, 150 mmol). The mixture is stirred for 3 h at 80 °C, then cooled down to room temperature and hydrolyzed with water (100 mL). After extraction of the aqueous layer with Et_2O (3 × 100 mL), the combined organic layers are successively washed with aqueous solution of HCl 1 M (15 mL), water (2 × 100 mL), and brine (2 × 100 mL), then dried over magnesium sulfate and concentrated. 2-Methylcyclohex-2-enone (5.09 g, 46.2 mmol) is obtained as a pure brown oil (yield: 92% over 2 steps). ^{1}H NMR (300 MHz, $CDCl_3$) δ ppm 1.76 (q, *J* = 1.8 Hz, 3H), 1.97 (pent, *J* = 6.2 Hz, 2H), 2.27–2.36 (m, 2H),

2.42 (dd, J = 6.5 Hz, 2H), 6.71–6.76 (bs, 1H); ^{13}C NMR (75 MHz, $CDCl_3$) δ ppm 16.1, 23.4, 26.1, 38.4, 135.7, 145.7, 200.1; MS (EI, 70 eV) *m/z*: 110 (M^+), 82 (M − CO, base), 67, 54.

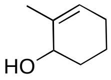

2-Methylcyclohex-2-enol 12 (CAS registry number: 20461-30-7)

To a solution of lithium aluminium hydride (712 mg, 18.8 mmol) in anhydrous THF (50 mL) under argon atmosphere is added dropwise 2-methylcyclohex-2-enone in anhydrous THF (25 mL). The mixture is stirred for 3 min at room temperature then slowly transferred into a separating funnel containing water (75 mL) and AcOEt (75 mL). The organic layer is washed with brine (2 × 50 mL), dried over magnesium sulfate then concentrated to provide the pure **12** (1.7 g, 15.2 mmol, 89%) as a pale yellow oil. ^{1}H NMR (300 MHz, $CDCl_3$) δ ppm 1.52–2.07 (m, 7H), 1.76 (d, J = 1.9 Hz, 3H), 3.97 (t, J = 4.0 Hz, 1H), 5.53 (bs, 1H); ^{13}C NMR (75 MHz, $CDCl_3$) δ ppm 18.2, 20.8, 25.5, 32.3, 68.5, 125.6, 135.4; IR (neat): 3440, 2932, 1704, 1633, 1453, 1190; MS (EI, 70 eV) *m/z*: 112 (M^+), 97 (M − CH_3, base), 69.

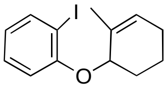

1-Iodo-2-(2-methylcyclohex-2-enyloxy)benzene (13)

Ether **13** is obtained using 2-iodophenol (2.69 g, 12.2 mmol), alcohol **12** (1.25 g, 11.1 mmol), triphenylphosphine (3.2 g, 12.2 mmol), and DIAD (2.42 mL, 12.2 mmol) in anhydrous THF (50 mL) following the Mitsunobu procedure. The pure **13** (2.36 g, 7.5 mmol, 68%) is isolated from the crude by flash chromatography on silica (10% of Et_2O in *n*-pentane) as a pale yellow oil. ^{1}H NMR (300 MHz, $CDCl_3$) δ ppm 1.55–1.65 (m, 1H), 1.74–1.86 (m, 2H), 1.84 (d, J = 1.7 Hz, 3H), 1.91–2.06 (m, 2H), 2.08–2.21 (m, 1H), 4.61 (bs, 1H), 5.74 (bs, 1H), 6.69 (td, J = 7.7, 1.4 Hz, 1H), 6.90 (dd, J = 8.3, 0.7 Hz, 1H), 7.28 (td, J = 8.4, 1.8 Hz, 1H), 7.78 (dd, J = 7.8, 1.6 Hz, 1H); ^{13}C NMR (75 MHz, $CDCl_3$) δ ppm 18.5; 21.3; 25.5; 28.2; 76.3; 88.4; 113.9; 122.5; 127.6; 129.4; 132.6; 139.8; 157.5; IR (neat): 3058, 2937, 1717, 1664, 1579, 1468, 1240, 1016 ; MS (EI, 70 eV) *m/z*: 314 (M^+), 220, 204, 95, 94 (base); Elemental analysis: calcd for $C_{13}H_{15}IO$: C, 49.70; H, 4.81; found: C, 49.47; H, 4.74.

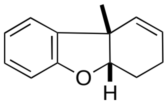

***cis*-9b-methyl-3,4,4a,9b-tetrahydrodibenzo[*b*,*d*]furan (14)**

The compound **14** is obtained using the ether **13** (314 mg, 1 mmol), $NiBr_2$bipy (75 mg, 0.2 mmol), and manganese powder (110 mg, 2 mmol) in anhydrous DMF (5 mL) following the carbonickelation procedure. The pure **14** (97 mg, 0.52 mmol, 52%) is isolated from the crude by

flash chromatography on silica (2% of Et_2O in *n*-pentane) as a colorless oil. ^1H NMR (300 MHz, $CDCl_3$) δ ppm 1.41 (s, 3H), 1.78–2.03 (m, 2H), 2.18–2.31 (m, 2H), 4.62 (t, *J* = 3.6 Hz, 1H), 5.51–5.56 (m, 1H), 5.68–5.75 (m, 1H), 6.79 (dd, *J* = 8.4, 1.0 Hz, 1H), 6.87 (td, *J* = 7.2, 0.9 Hz, 1H), 7.11 (d, *J* = 7.2 Hz, 1H), 7.12 (td, *J* = 6.6, 1.2H z, 1H); ^{13}C NMR (75 MHz, $CDCl_3$) δ ppm 19.4, 23.3, 25.1, 44.4, 87.8, 110.0, 120.7, 122.9, 125.5, 128.1, 132.1, 135.8, 158.9; NMR 2D NOESY: correlation between 1.41 (s, 3H) and 4.62 (t, *J* = 3.6 Hz, 1H); IR (neat): 3018, 2956, 1595, 1474, 1232, 1039 cm^{-1}; MS (CI) *m/z*: 186 (M^+), 171 (M − Me, base), 143, 128; HRMS (EI): calcd for (M^+) $C_{13}H_{14}O$: 186.1045, found: 186.1049.

Pd process in Fukuyama conditions: To a solution of **13** (478 mg, 1.52 mmol) in CH_3CN (10 mL) under argon is added tri-*o*-tolylphosphine (93 mg, 0.31 mmol), triethylamine (0.42 mL, 3.04 mmol) and $Pd_2(dba)_3$ (70 mg, 0.08 mmol). The black mixture is heated under reflux for 1 h then cooled down to rt before adding TBAF (1 M/THF, 1.8 mL, 1.8 mmol). After stirring for 1 h, the solution is hydrolyzed with saturated aqueous ammonium chloride (10 mL) and filtered on Celite, and the aqueous layer is extracted with AcOEt (3 × 10 mL). The combined organic layers are washed with brine (10 mL) then dried over magnesium sulfate and concentrated. Purification of the crude by flash chromatography (pentane/Et_2O 98/2) provides a mixture of the 3 inseparable isomers **14, 15** and **16** (232mg, 1.25 mmol, 82%) in the ratio 60/15/25 as a colorless oil.

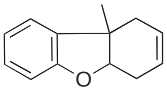

9b-Methyl-1,4,4a,9b-tetrahydrodibenzo[*b*,*d*]furan (15)

^1H NMR (300 MHz, $CDCl_3$) δ ppm 1.38 (s, 3H), 2.24–2.77 (m, 2H), 2.27–2.42 (m, 1H), 2.50–2.62 (m, 1H), 4.59–4.62 (m, 1H), 5.83–5.84 (m, 2H), 6.72 (d, *J* = 7.8 Hz, 1H), 6.80–6.90 (m, 1H), 7.05–7.14 (m, 2H); MS (CI) *m/z*: 186 (M^+), 171 (M − Me, base), 143, 128; HRMS (EI): calcd for (M^+) $C_{13}H_{14}O$: 186.1045; found: 186.1049.

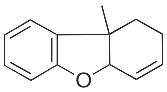

9b-Methyl-1,2,4a,9b-tetrahydrodibenzo[*b*,*d*]furan (16)

^1H NMR (300 MHz, $CDCl_3$) δ ppm 1.31 (s, 3H), 1.83–1.90 (m, 1H), 1.99–2.04 (m, 1H), 2.10–2.24 (m, 2H), 4.45–4.57 (m, 1H), 5.90–5.95 (m, 1H), 6.02–6.10 (m, 1H), 6.77 (d, *J* =8.4 Hz, 1H), 6.80–6.90 (m, 1H), 7.05–7.14 (m, 2H); MS (CI) *m/z*: 186 (M^+), 171 (M − Me, base), 143, 128; HRMS (EI): calcd for (M^+) $C_{13}H_{14}O$: 186.1045; found: 186.1045.

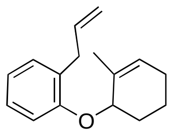

1-Allyl-2-(2-methylcyclohex-2-enyloxy)benzene (17)

The compound **17** is obtained using the ether **13** (233 mg, 0.74 mmol), AllylOAc (160 µL, 1.48 mmol), $NiBr_2$bipy (75 mg, 0.2 mmol), manganese powder (110 mg, 2 mmol) in anhydrous DMF (5 mL) following the carbonickelation procedure. The pure **17** (90 mg, 0.39 mmol, 53%) is isolated from the crude by flash chromatography on silica (*n*-pentane) as a colorless oil. ^{1}H NMR (300 MHz, $CDCl_3$) δ ppm 1.52–1.59 (m, 1H), 1.67–1.78 (m, 2H), 1.78–1.82 (m, 3H), 1.92–2.03 (m, 2H), 2.08-2.17 (m, 1H), 3.41 (d, *J* = 6.5 Hz, 2H), 4.62 (bs, 1H), 5.03 (dd, *J* =10.2, 1.6 Hz, 1H), 5.06, dd, *J* = 17.0, 1.6 Hz, 1H), 5.71 (bs, 1H), 6.00 (ddt, *J* = 17.0, 10.2, 6.6 Hz, 1H), 6.89 (t, *J* = 7.5 Hz, 1H), 6.93 (d, *J* = 8.2 Hz, 1H), 7.16 (d, *J* = 7.5 Hz, 1H), 7.18 (t, *J* = 7.8 Hz, 1H); ^{13}C NMR (75 MHz, $CDCl_3$) δ ppm 18.3, 21.2, 25.5, 27.9, 34.5, 73.8, 112.5, 115.3, 120.2, 126.9, 127.1, 129.7, 130.0, 133.0, 137.2, 156.0; MS (CI) *m/z*: 229 (MH^+), 214, 177, 135 (base), 119, 107, 94; Elemental analysis: calcd for $C_{16}H_{20}O$: C, 84.16; H, 8.83; found: C, 83.85; H, 8.67.

References

1. Uchino, M.; Asagi, K.; Yamamoto, A.; Ikeda, S. *J. Organomet. Chem.* **1975**, *84*, 93–103.
2. Dunach, E.; Périchon, J. *J. Organomet. Chem.* **1988**, *352*, 239–246.
